# Oscillatory Dynamics and Energy Transport in Twisted Magnetic Flux Tubes of the Solar Photosphere

**DOI:** 10.1007/s11207-026-02652-y

**Published:** 2026-04-20

**Authors:** Adel S. Alanezy, Suzana S. A. Silva, Istvan Ballai, Gary Verth, Viktor Fedun

**Affiliations:** 1 Plasma Dynamics Group, School of Mathematical and Physical Sciences, Hicks Building, Hounsfield Road, Sheffield, S3 7RH UK; 2 Department of Mathematics, Faculty of Science, Islamic University of Madinah, Madinah, Saudi Arabia; 3 Plasma Dynamics Group, School of Electrical and Electronic Engineering, University of Sheffield, Sheffield, S1 3JD UK

**Keywords:** Decomposition methods, Magnetic flux tubes, Waves, Magnetic fields

## Abstract

We investigate the oscillatory behaviour of the footpoints of twisted magnetic flux tubes in the solar photosphere. We identify the dominant magnetohydrodynamic (MHD) wave modes present in these waveguides and assess their role in energy transport. Using vector magnetograms from the Solar Dynamics Observatory/Helioseismic and Magnetic Imager Space-weather HMI Active Region Patches (SDO/HMI SHARP) series of active region 11158, the footpoints of twisted flux tubes are identified as convex local maxima of the Integrated Average Current Deviation (IACD) field, which highlights regions of enhanced magnetic twist and current concentration. To study the waves propagating in these structures, we apply the Spectral Proper Orthogonal Decomposition (SPOD) method, which separates complex spatio-temporal data into oscillatory patterns and their characteristic frequencies. Our analysis shows that the footpoints of the twisted flux tubes support both kink and sausage MHD modes, with oscillations detected across multiple diagnostics, including IACD, the vertical magnetic field, and the vertical Poynting flux. The coexistence of these modes suggests nonlinear interactions or mode coupling within the twisted magnetic structures. These twisted flux tubes act as magnetic waveguides that modulate the vertical transport of energy between the photosphere and higher atmospheric layers. The inferred upward Poynting fluxes ($10^{5}$ – $10^{6}~\mathrm{W\,m^{-2}}$) indicate that such twisted magnetic features may contribute to localised chromospheric heating.

## Introduction

The investigation of twisted magnetic flux tubes as waveguides is an essential research topic in solar physics and magnetohydrodynamics (MHD). These structures act as pathways for various wave modes, including kink, torsional Alfvén, and sausage waves (e.g. Erdélyi and Fedun 2006). The properties of twisted magnetic flux tubes make them effective waveguides due to their geometric and magnetic configurations. Williams, Berger, and Pontin (2016) examined the nonlinear evolution of twists in magnetic shock tubes. They showed that a highly twisted magnetic flux tube facilitates sub-Alfvénic mass flows. These flows significantly influence the magnetic structure and wave propagation. Similarly, Fan (2009) highlighted the emergence of twisted flux tubes in the solar atmosphere. Their work suggests that nonlinear torsional Alfvén waves are generated, transporting twist from the interior to the corona.

Numerous studies have reported on the effects of twist on wave propagation. Erdélyi and Fedun (2006) focused on sausage MHD waves in incompressible flux tubes with twisted magnetic fields. They derived a dispersion relation that aids in studying surface and body waves. Such studies are essential for understanding dynamics within twisted flux tubes throughout the solar atmosphere. Erdélyi and Fedun (2007) expanded this work to compressible magnetically twisted flux tubes. They noted that the twist affects wave properties, including damping and resonance conditions. Zhelyazkov and Zaqarashvili (2012) investigated Kelvin-Helmholtz instability of kink waves in twisted magnetic flux tubes. They revealed how wave amplification can occur when these tubes interact with surrounding plasma. Their research suggests that interactions between twisted flux tubes and external magnetic fields can lead to observable instabilities. These instabilities may affect wave propagation and energy transfer in the solar atmosphere. This instability highlights the capacity of twisted tubes to act as dynamic waveguides that can amplify certain wave modes under specific conditions. This interplay between wave propagation and flux tube stability has been addressed in various studies. For example, Karami and Barin (2009) provided a comprehensive analysis of how twisted magnetic fields influence the oscillation characteristics of coronal loops. They showed that damping rates for MHD waves are significantly affected by the twisted structure, which governs oscillation patterns and energy dissipation rates. This indicates that twist can modify the natural frequencies of oscillations in the coronal environment. As a result, twist affects how energy is transported from the solar interior to the corona. Török and Kliem (2003) researched coronal magnetic flux tubes, emphasising the evolution and maintenance of their magnetic structure during wave propagation. Their work underscores the importance of carefully modelling twisted configurations to understand their waveguide properties.

A crucial aspect to study twisted magnetic flux tubes as waveguides in observations is automatically identifying such structures. One fundamental method for detection involves analysing their topological characteristics. MacTaggart, Prior, and Raphaldini (2021) provided evidence for the emergence of pre-twisted magnetic flux tubes by investigating a robust topological quantity called magnetic winding. This approach allows researchers to detect emerging magnetic topologies, even as the magnetic field undergoes significant deformations in the solar environment. The magnetic winding complements magnetic helicity and provides unique insights into the flux-tube topology during emergence. Recent work by Wagner et al. (2024) identifies 3D twisted magnetic flux tubes based on key parameters, such as the twist parameter and current density. In this study, we identify twisted magnetic field structures using the Integrated Averaged Current Deviation (IACD; Rempel et al. 2019), which can automatically locate the footpoints of the most relevant twisted magnetic flux tubes. Once these structures are identified, the next challenge is to characterise the wave modes that propagate through them. Observational identification of such wave modes requires methods capable of disentangling multiple overlapping oscillations in both space and time. To address this, we employ modal decomposition techniques, which provide a powerful framework for uncovering coherent structures in complex datasets. By decomposing spatio-temporal fields into orthogonal modes, these approaches reveal the dominant patterns that govern plasma dynamics. Proper Orthogonal Decomposition (POD) and Dynamic Mode Decomposition (DMD) have been successfully applied to high-resolution solar observations (Aldhafeeri et al. 2021), helping to identify MHD wave signatures in magnetic structures such as sunspots. These techniques combine spatial and temporal information, offering an alternative to traditional Fourier analysis that more effectively isolates physically meaningful oscillations.

The primary aim of this work is to characterise the oscillatory modes supported by twisted magnetic flux tubes in an active region and to quantify their associated energy fluxes. In particular, we investigate whether consistent frequencies appear across different diagnostics such as IACD, vertical magnetic field, and Poynting flux to assess the presence of physical oscillations that couple magnetic and energetic processes. By linking mode identification with energy-transport estimates, this study aims to clarify how small-scale twisted magnetic flux tubes contribute to the coupling between the photosphere and higher layers of the solar atmosphere. To achieve this, we apply an advanced version of POD, the Spectral Proper Orthogonal Decomposition (SPOD; Sieber, Paschereit, and Oberleithner 2016), to analyse oscillatory processes in selected photospheric footpoints of twisted magnetic flux tubes within an active region. The paper is organised as follows. The IACD method, SPOD framework, and dataset are described in the Methodology section; the identified twisted flux tubes and detected wave modes are presented in the Results; and the main findings and conclusions are summarised in the final section.

## Methodology

### SHARP Data

Our analysis is focused on the active region (AR) 11158, as catalogued by the National Oceanic and Atmospheric Administration (NOAA). This is a well-studied region in the literature, and it was used to investigate the rapid restructuring of the photospheric fields during an X2.2 flare, as well as in numerous flare-forecasting and magnetic-field evolution case studies (see, e.g., Schrijver et al. 2011; Beauregard, Verma, and Denker 2012; Wang et al. 2012; Raja Bayanna et al. 2014). We used the ARTop^1^(Alielden et al. 2023), an open software to download SHARP (Space-weather HMI Active Region Patches; Bobra et al. 2014) magnetograms. The velocity field components were also recovered by ARTop, which uses the Differential Affine Velocity Estimator for Vector Magnetograms (DAVE4VM) algorithm (Schuck 2008). DAVE4VM is employed to recover the photospheric velocity field from time-series vector magnetogram observations. DAVE4VM infers the plasma flow by assuming that, within a small spatial window (20 by 20 pixels in our analysis), the velocity field can be approximated by a first-order affine transformation. This local affine model is constrained by the induction equation under the ideal MHD assumption, which relates temporal changes in the magnetic field to the curl of the electric field and, consequently, to plasma motions. By minimising the discrepancy between the observed magnetic field evolution and that predicted by the affine model, DAVE4VM derives both the horizontal and vertical components of the velocity consistent with the observed changes in all three components of the magnetic field. This approach allows for a physically consistent estimation of the local flow field even in regions of complex magnetic structure.

The structure and evolution of the magnetic field associated with this AR have been analysed in detail by Wang et al. (2012) using SDO/HMI data. Their research provided evidence of rapid and permanent changes in the photospheric magnetic field associated with major flaring activity in this AR. In particular, their study revealed a significant evolution in the magnetic topology, with a considerable enhancement of the horizontal magnetic field component, by approximately 400 G, equivalent to a 30% increase over its pre-flare value, within just 30 minutes. This enhancement occurred in close temporal proximity to the hard X-ray emission of the flare and was accompanied by a pronounced increase in magnetic shear and restructuring of the magnetic field, indicating a rise in non-potentiality near the surface. Such restructuring of the magnetic field and the localised increase in horizontal field strength and shear provide a favourable environment for the development and persistence of twisted magnetic structures.

**Figure 1:**
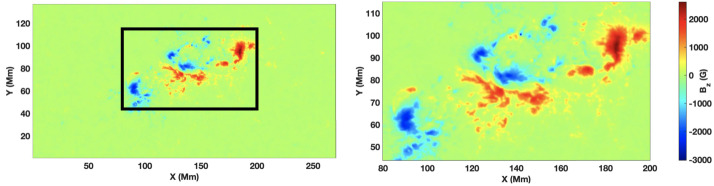
Field-of-view map of the vertical magnetic field component, $B_{z}$, derived from HMI magnetograms. The left panel displays the complete field of view, while the black box outlines the AR subfield selected for analysis. The right panel shows a magnified view of this region, which represents the primary region of interest used throughout this work. All quantitative analyses presented in this study are based on this cropped domain.

The complete field of view used in our analysis is shown in Figure 1 (showing the vertical component of the magnetic field), with the black box highlighting the AR we are analysing (left panel). On the right, we present a zoomed-in view of the region.

### Integrated Averaged Current Deviation

We identified the footpoints of the twisted magnetic flux tubes by computing the IACD from the SHARP magnetic field. The IACD quantifies spatial deviations of the current density, and it is defined as (see, e.g., Rempel et al. 2019)

1 $$ \mathrm{IACD}_{s_{0}}^{s_{0}+\xi}(\mathbf{x}_{0}, t) := \int _{s_{0}}^{s_{0}+ \xi} \left |\mathbf{J}(\mathbf{x}(s), t) - \langle \mathbf{J}(t) \rangle \right | ds, $$

where the current density $\mathbf{J}$ is given by $\mathbf{J} = (\nabla \times \mathbf{B})/\mu _{0}$, with $\mu _{0}$ denoting permeability of free space. The vector $\mathbf{x}$ indicates spatial position, and $\langle~\rangle $ denotes the spatial average of the current density at a given time $t$. The integration is performed along a magnetic field line, beginning from a reference point $\mathbf{x}_{0}$, and $ds$ represents an infinitesimal arc-length segment along that line.

We perform the integration by considering only the horizontal components of the magnetic field. Thus, at each time frame, we numerically integrate the parametric field-line equation

$$ \frac{d\mathbf{x}}{ds} = \mathbf{B}(\mathbf{x}(s), t_{0}), $$

where $\mathbf{x}(s)$ denotes the position along the field line, and $s$ is a dimensionless parameter related to the arc length $l$ through

$$ dl = |\mathbf{B}|\, ds. $$

The integration is initialised at $\mathbf{x}(s_{0}) = \mathbf{x}_{0}$, defining an initial-value problem evaluated at a fixed time. For each grid point, we integrate the trajectories along the field lines using a fourth-order Runge-Kutta scheme, combined with third-order spatial interpolation of $\mathbf{B}$ on the discrete grid. We adopt an integration length of $\xi = 30$ and a step size of $ds = 0.1$.

Twisted magnetic flux tubes are identified through local maxima in the IACD field, where enhanced current deviation forms convex, coherent structures. In the present context, we define a coherent structure as a magnetic entity with a well-defined cross-section. Since SHARP data provide only the photospheric vector field, these convex IACD maxima correspond to the 2D photospheric footprints of fully three-dimensional twisted flux tubes. Thus, the detected structures trace the lower-boundary anchoring of a 3D magnetic system whose geometry and twist likely extend upward into the chromosphere and corona. Consequently, the motion and perturbations of the IACD features reflect the dynamics of the underlying twisted magnetic flux tubes. In other words, analysing the temporal evolution of these convex IACD maxima enables us to investigate and track the motion and oscillatory behaviour of the twisted magnetic flux tubes. For instance, the increase in time in the area of the coherent IACD features indicates the expansion of the twisted magnetic flux tube. At the same time, a weakening of the IACD signal within such structures suggests that the magnetic structures are becoming less twisted.

### Spectral Proper Orthogonal Decomposition

To identify wave structures supported by the twisted flux tubes, we analyse the time evolution of the IACD-identified core of each tube by applying SPOD to the corresponding region of interest (ROI). SPOD extracts dominant spatial modes from time-resolved data. Given a field variable $q(\mathbf{x}, t)$, its fluctuation is defined as

2 $$ q'(\mathbf{x}, t) = q(\mathbf{x}, t) - \langle q(\mathbf{x}) \rangle , $$

where $\langle~\rangle $ denotes the temporal mean, $t$ represents time, and $\mathbf{x}$ is the spatial coordinate vector.

The fluctuating field can be expressed as a modal expansion:

3 $$ q'(\mathbf{x}, t) = \sum _{n=1}^{N} \phi ^{(n)}(\mathbf{x})\, a^{(n)}(t), $$

where $N$ is the total number of snapshots, $\phi ^{(n)}(\mathbf{x})$ are the spatial modes, and $a^{(n)}(t)$ are the corresponding temporal coefficients. The superscript $(n)$ denotes the mode index.

The modal basis is obtained by solving the eigenvalue problem

$$ C \xi = \Lambda \xi , $$

where $C$ is the covariance matrix, $\xi $ is the eigenvector matrix, and $\Lambda $ is a diagonal matrix containing the eigenvalues. For our temporal sequence of 2D data, the temporal covariance matrix elements are defined as

4 $$ C_{t_{1},t_{2}} = \frac{1}{N} \int _{\Omega} q'(\mathbf{x}, t_{1})\, q'( \mathbf{x}, t_{2})\, d\mathbf{x}, $$

with $t_{1}$ and $t_{2}$ denoting distinct time indices.

Unlike the classical POD, SPOD incorporates a spectral filtering step applied to the covariance matrix, as discussed by Sieber, Paschereit, and Oberleithner (2016). The spatial modes are obtained as

5 $$ \phi ^{(n)}(\mathbf{x}) = \frac{1}{\lambda _{n} N} \sum _{j=1}^{N} \xi _{j,n}\, q'(\mathbf{x}, t_{j}), $$

where $\lambda _{n}$ is the $n$th eigenvalue of $\Lambda $. The corresponding temporal coefficients are given by

6 $$ a^{(n)}(t_{j}) = \sqrt{N \lambda _{n}}\, \xi _{j,n}. $$

The time-dependent coefficients $a^{(n)}(t)$ describe the temporal evolution of each mode, facilitating the identification of wave phenomena and their dynamical characteristics, such as frequency, growth, or decay.

SPOD is a very useful tool for decomposing inhomogeneous unsteady flows, revealing the spatial structure and temporal evolution of each mode based on their contributions to the overall energy content of a signal, and filters modes so that for most cases each spatial mode has only one frequency.

## Results

### Identification of Photospheric Footpoints of Twisted Magnetic Flux Tubes

**Figure 2:**
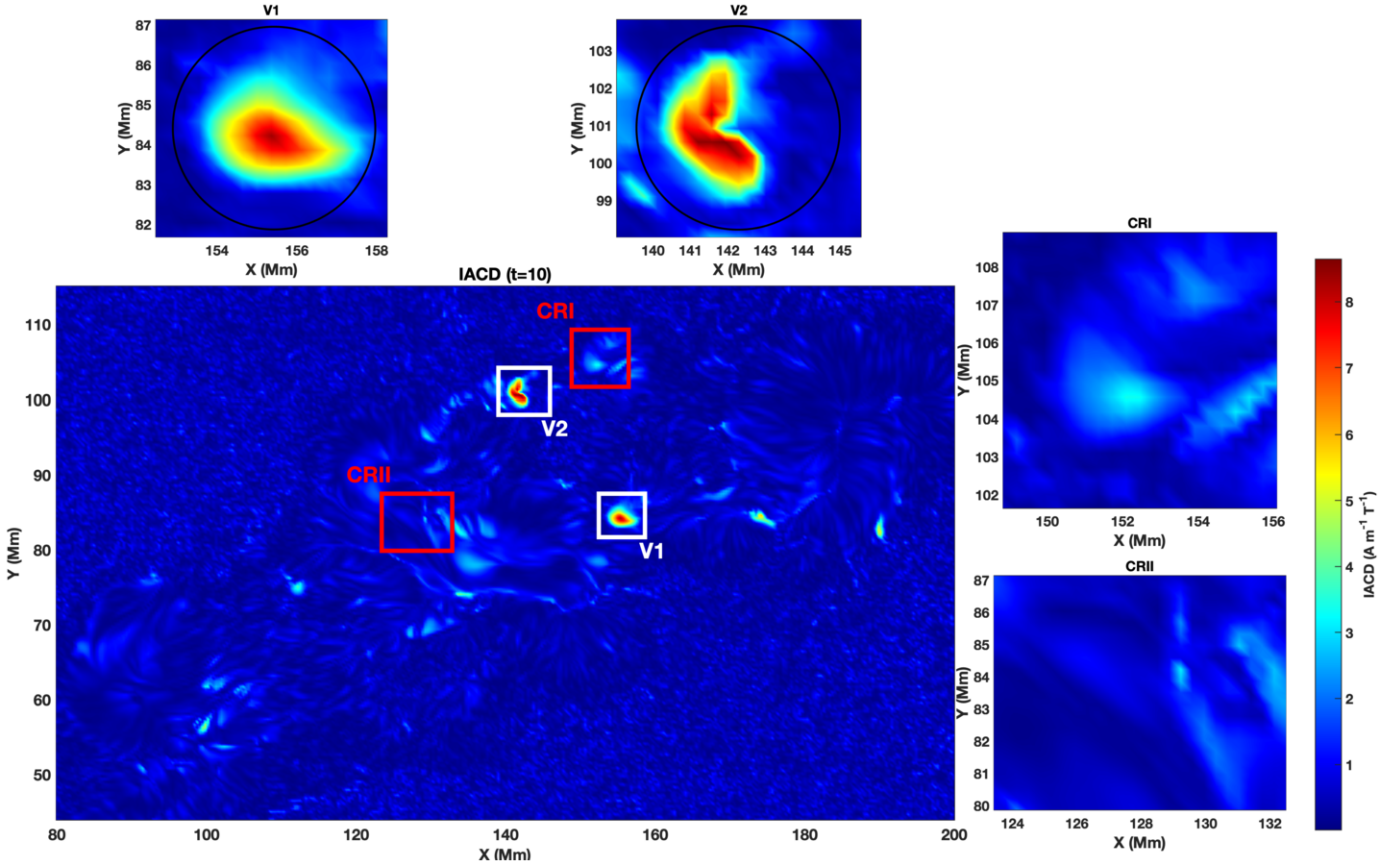
The IACD intensity distribution calculated with the use of Eq. 1 for AR 11158 on 2011-02-13 at 19:00:00 UT is shown. The central panel shows the FOV with two selected twisted magnetic flux tubes (V1 and V2) highlighted by white boxes, as well as two control regions (here, and in all subsequent plots, denoted by CRI and CRII, highlighted within the red boxes). The two top panels show the zoomed-in regions containing the twisted flux tubes V1 and V2. The side panels show the zoomed-in regions containing the control regions CRI and CRII.

Figure 2 displays the full field of view (FOV) encompassing the AR NOAA 11158, where two prominent twisted magnetic structures, labelled V1 and V2, were detected as convex-shaped concentrations of the IACD. The top two sub-panels show magnified views of these regions. The twisted magnetic flux tube V1 exhibits a compact and nearly circular morphology, whereas V2 initially displays a more elongated and asymmetric geometry. We select those structures as they were both the largest structures that persisted coherently over multiple consecutive frames. The two panels on the right depict the control regions used later for our analysis.

**Figure 3:**
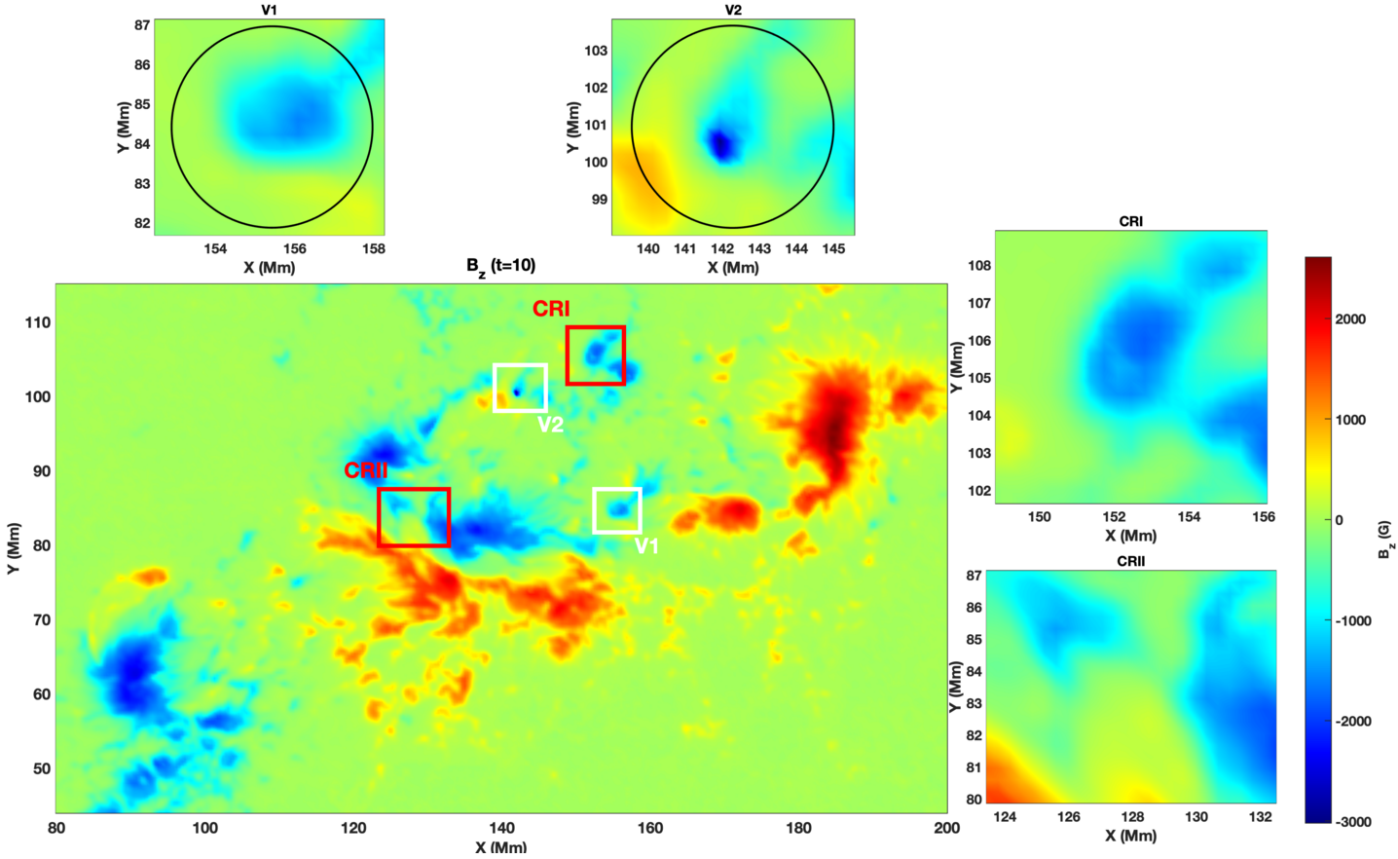
Structure of the vertical component of the magnetic field in the selected domain. The central panel shows the vertical magnetic field component, $B_{z}$, of AR 11158 on 2011-02-13 at 19:00:00 UT, while the top panels display the zoomed-in views of the regions where the twisted magnetic flux tubes were identified (V1 and V2), as well as the two control regions (CRI and CRII).

The corresponding vertical magnetic field component, $B_{z}$, is presented in Figure 3. The central panel shows the complete $B_{z}$ map on 2011-02-13 at 19:00:00 UT, while the top panels provide zoomed views centred on V1 and V2, respectively. Both footpoints of the twisted magnetic flux tubes show compact magnetic flux concentrations, and in each case, the field is predominantly directed downward. This pattern suggests that V1 and V2 represent the footpoints of separate twisted magnetic loops. The comparison between the IACD and $B_{z}$ maps confirms that both flux tubes coincide with zones of enhanced current deviation and magnetic flux concentration.

### Wave Identification

To investigate the nature of the perturbations within the selected twisted magnetic flux tubes, we applied SPOD to the datasets of both isolated structures to isolate and characterise wave modes. SPOD ranks the modes in terms of their contribution to the observed perturbations in the signal. A given SPOD mode is identified as a wave mode if both its temporal and spatial characteristics exhibit signatures consistent with wave behaviour. Specifically, the temporal coefficient must display clear periodic oscillations about zero, while the spatial structure should exhibit patterns consistent with the expected wave signatures for circular or elliptical waveguides. Modes that do not satisfy both criteria are not considered physically meaningful waves. Those other modes can be either transients, other dynamics, noise, or spurious signals (see, e.g., Albidah et al. 2021; Jafarzadeh et al. 2024). Therefore, these will not be taken into account.

**Figure 4:**
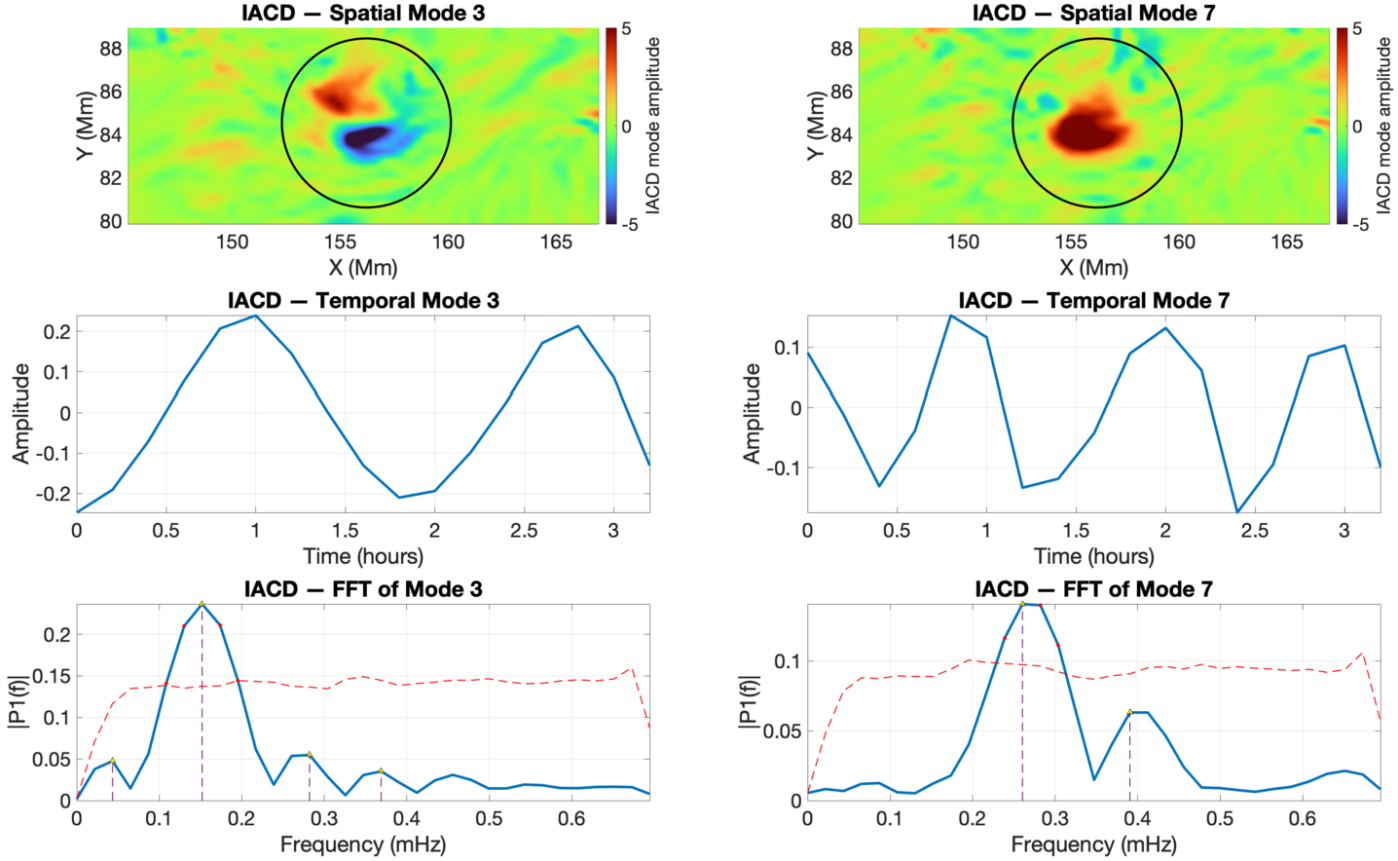
Wave mode identification by SPOD for the twisted flux tube V1 during the interval 2011-02-13 18:00:00 UT – 21:12:00 UT. The top row shows the spatial structures of modes 3 (kink) and 7 (sausage). The middle row presents the corresponding temporal coefficients. The bottom row shows the Fourier spectra of the temporal modes.

The SPOD analysis was restricted to the time interval where the IACD field displays coherent regions of maxima. As these regions evolve, they eventually lose coherence and fragment toward the end of their lifetime, which can influence the accuracy of mode identification. To analyse the cross-sectional evolution of the twisted magnetic flux tubes, we used 16 consecutive frames for V1 (2011-02-13 18:00:00 UT – 21:12:00 UT) and the first 19 consecutive frames for V2 (2011-02-13 19:00:00 UT – 22:48:00 UT). Figure 4 shows the SPOD modes 3 and 7 recovered from V1. The spatial modes are shown in blue for negative and in red for positive values of the perturbations. The second row shows the temporal variation of the time coefficients, while the third row displays the fast Fourier transform (FFT) of the time coefficients. The red dashed line marks the 95% confidence level obtained from a permutation test (Jafarzadeh et al. 2024), showing the threshold above which spectral peaks can be considered statistically significant and not due to random fluctuations. The black circles shown in the first row of Figure 4 indicate the regions within the selected ROI where V1, the footpoint of a twisted magnetic flux tube, moves during the analysed time interval. The centre of these circles corresponds to the pixel of maximum mean of the IACD intensity.

The nature of the identified mode is determined based on the similarities between the spatial structure of the mode and the theoretical predictions of a perfectly cylindrical waveguide (Edwin and Roberts 1983). Although we are clearly not dealing with waveguides whose cross-section is a perfect circle, earlier investigations by Aldhafeeri et al. (2021), Albidah et al. (2021) have shown that deviations of the cross-section from a circular shape affect primarily higher order modes, while fundamental and low order modes remain largely unchanged. The temporal coefficients of the first two empirical modes for V1 reveal harmonic perturbations with peak frequencies at 0.15 mHz and 0.26 mHz, respectively. We should mention here that these frequencies (and all subsequent) do not necessarily correspond to real physical eigenmodes, as these values are strongly influenced by the cadence of the observational data we are using. Instead, the frequencies are used to identify correlated changes in perturbations and evidence of oscillatory patterns in various physical quantities. That is also the reason why we will not perform any detailed analysis of the consequences of observed frequencies (e.g. seismological analysis).

The first mode (first column) is due to the movement of the centre of mass corresponding to a kink mode (in a perfectly cylindrical waveguide, these modes correspond to an azimuthal wavenumber $m=1$). The second identified mode is due to the radial expansion and contraction of the flux tube in all directions, thus representing a sausage mode (the azimuthal wave number, $m=0$), when compared to the cylindrical flux tube model. We should note that the oscillatory movements seen in the tube cross-sections may not perfectly align with the expected movements for the eigenmodes of a cylindrical flux tube (that exhibit perfect symmetry, both in terms of shape and oscillations). This discrepancy can be attributed not only to the irregular, non-circular shapes of the observed waveguides but also to the presence of asymmetric oscillations in the observations or local inhomogeneities in density and/or magnetic field, as evidenced by the study by Asiri et al. (2024). This variation, however, does not significantly impact the overall agreement or correlation between the observed and theoretical wave modes. In fact, the identification of each MHD wave mode is determined by the best agreement obtained, indicating the most accurate match between them.

**Figure 5:**
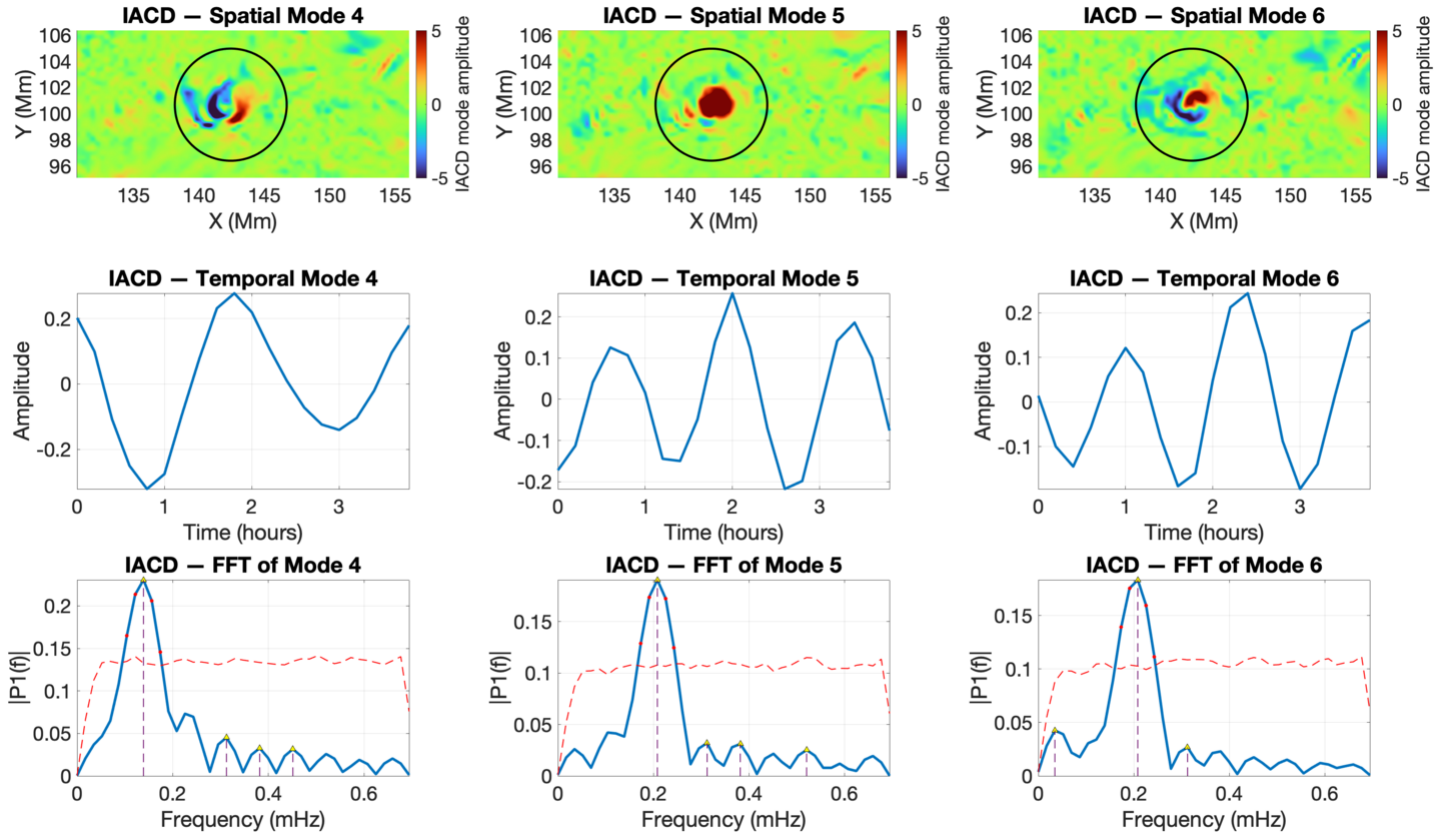
Wave mode identification by SPOD for the twisted flux tube V2 during the interval 2011-02-13 19:00:00 UT – 22:48:00 UT. The top row shows the spatial structures of modes 4 (kink), 5 (sausage), and 6 (helical). The middle row presents the corresponding temporal coefficients. The bottom row shows the Fourier spectra of the temporal modes.

For the V2 footpoint of the twisted magnetic flux tube, the SPOD analysis of the IACD field reveals that the structure supports at least three well-defined oscillatory modes (Figure 5). Mode 4 corresponds to a kink oscillation with a frequency of 0.14 mHz, while mode 5 displays a symmetric spatial pattern characteristic of a sausage mode, associated with a frequency of 0.2 mHz. Mode 6 also oscillates at approximately 0.2 mHz, but its spatial structure departs from the standard kink morphology and instead exhibits a slight “twist” or “yin–yang” pattern. This deformation likely results from interactions with local rotational plasma flows (see, e.g., Maxworthy, Hopfinger, and Redekopp 1985), producing a helical or bending-type mode often described in fluid-dynamical contexts. Finally, we note that the oscillation frequencies retrieved in this study fall in an ultra-low frequency range. This is a direct consequence of the limited temporal cadence of the HMI vector data ($\approx 12$ minutes), which restricts the detectable frequencies to values well below those expected for typical photospheric MHD waves. Higher-frequency oscillations, including the canonical three- and five-minute bands, may well be present in these structures but cannot be resolved with the available sampling. Thus, while the spatial patterns identified by our POD-based spectral decomposition are consistent with genuine MHD eigenmodes of the twisted flux tubes, the corresponding frequencies should be interpreted as low-frequency aliases imposed by the observational cadence rather than as physical eigenfrequencies of the system.

### Energy Transport by Twisted Magnetic Flux Tubes

To investigate the energy transport by the analysed twisted flux tubes, we computed the Poynting flux defined in the MHD approximation as (Shelyag et al. 2011; Silva et al. 2024):

7 $$ {\mathbf{S}}=\frac{1}{4\pi}({\mathbf{B}}\times {\mathbf{v}})\times {\mathbf{B}}, $$

where ${\mathbf{B}}$ and ${\mathbf{v}}$ are the magnetic field and plasma flow vectors. The vector ${\mathbf{S}}$ describes the direction in which magnetic energy is flowing, and its magnitude provides the rate per unit area at which the energy crosses a surface. Taking the coordinate system such that the $z$-axis points perpendicular to the solar surface, the Poynting flux vector can be decomposed into its horizontal ($h$) and vertical ($z$) components using

$$ {\mathbf{S}}_{h}=(S_{x},S_{y},0), \quad S_{x}=\frac{1}{4\pi}\left [v_{x}(B_{y}^{2}+B_{z}^{2})-B_{x}(v_{y}B_{y}+v_{z}B_{z}) \right ], $$

$$ S_{y}=\frac{1}{4\pi}\left [v_{y}(B_{z}^{2}+B_{x}^{2})-B_{y}(v_{x}B_{x}+v_{z}B_{z}) \right ], $$

$$ {S}_{z}=\frac{1}{4\pi}\left (v_{z}B_{h}^{2}-({\mathbf{v}}_{h}\cdot {\mathbf{B}}_{h})B_{z} \right ), $$

where ${\mathbf{v}}_{h}=(v_{x},v_{y},0)$ and ${\mathbf{B}}_{h}=(B_{x},B_{y},0)$. In the context of photospheric magnetic flux tubes, the vertical component of the Poynting flux ($S_{z}$) is the most relevant diagnostic tool, because it quantifies the energy transported along the magnetic field lines, i.e. between the photosphere and higher atmospheric layers. The horizontal components ($S_{x}$ and $S_{y}$) mainly describe lateral energy redistribution within the same layer and are highly susceptible to cancellation and noise in structured magnetic environments. Since the identified flux tubes act as vertical MHD waveguides supporting kink and sausage oscillations, $S_{z}$ directly traces the propagation of wave energy within these structures, providing a measure of their contribution to coupling between the photosphere and higher regions in the solar atmosphere.

**Figure 6:**
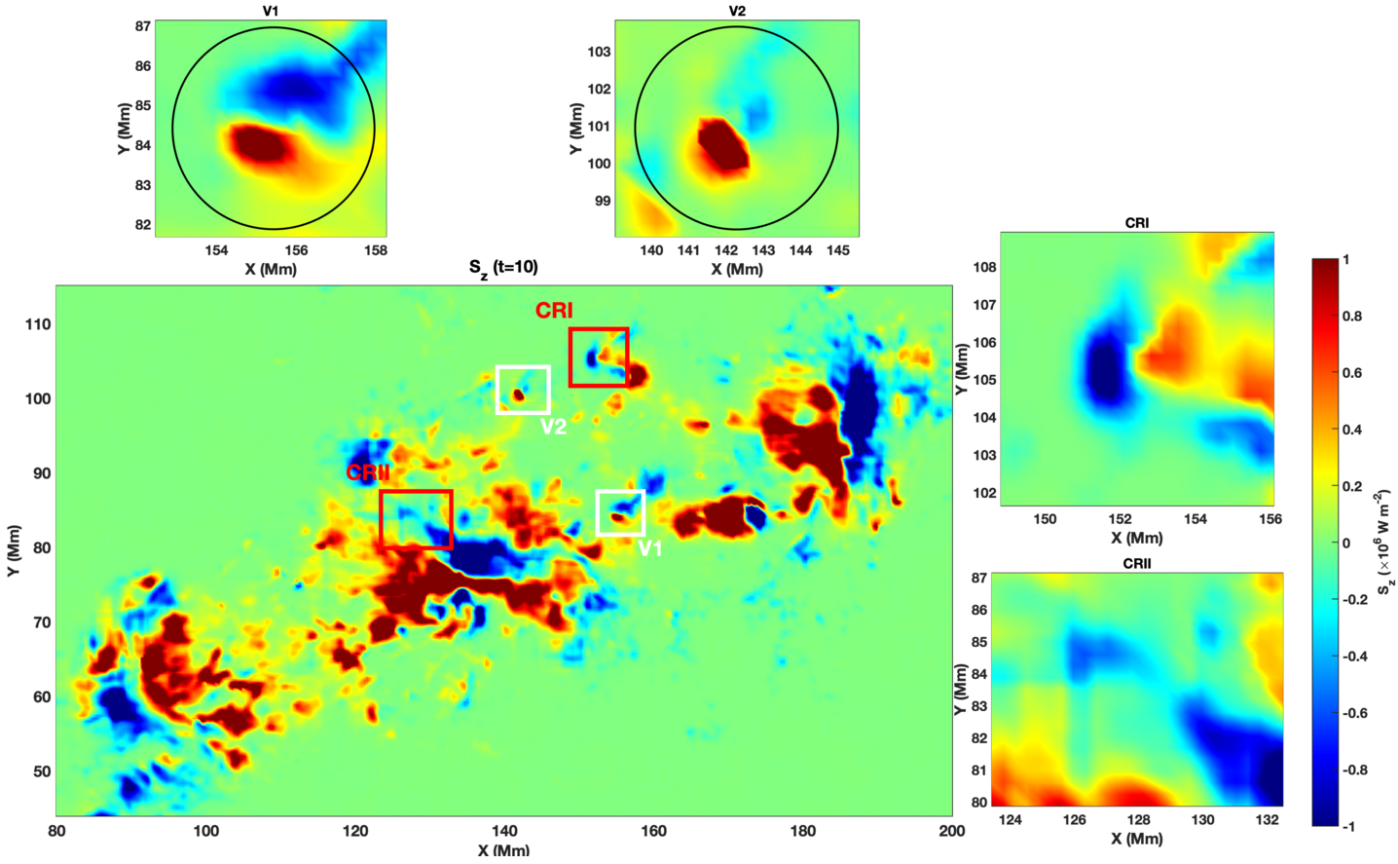
Vertical component of the Poynting flux. The central panel shows the full field of view of $S_{z}$ on 2011-02-13 at 18:00:00 UT, with the top panels displaying the zoomed-in regions containing the flux tubes V1 and V2, as well as the two control regions. The color scale represents $S_{z}$ in units of $10^{6}$ W m^−2^ s^−1^.

Using the components of the velocity and magnetic fields of AR11158, we calculated the vertical component of the Poynting vector as shown in Figure 6. Positive $S_{z}$ indicates upward energy transport from lower atmospheric layers, while negative $S_{z}$ signifies downward-directed energy flux. We again applied the SPOD method to examine the temporal and spatial evolution of the vertical Poynting flux component within the regions encompassing the two identified footpoints of twisted magnetic flux tubes. The same time intervals are used as before: 18:00:00 UT – 21:12:00 UT for V1 and 19:00:00 UT – 22:48:00 UT for V2, on 2011-02-13.

**Figure 7:**
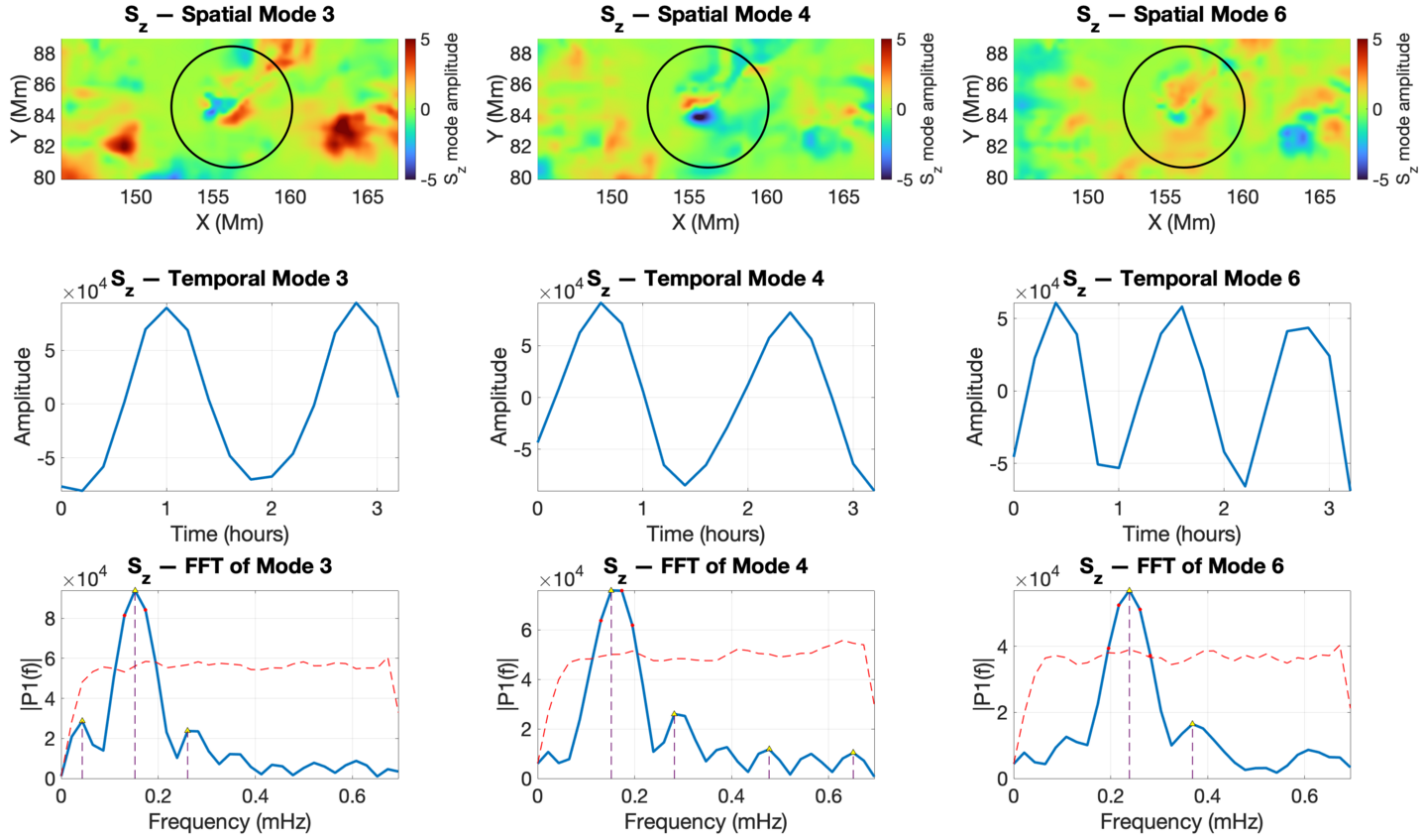
SPOD analysis of the vertical Poynting flux ($S_{z}$) in the region of the twisted magnetic flux-tube footpoint V1 during the interval 2011-02-13 18:00:00 UT – 21:12:00 UT. Top row: spatial SPOD modes; middle row: corresponding temporal coefficients; bottom row: FFT spectra of the temporal modes showing the dominant frequencies with 95% confidence threshold.

For the structure V1, the results of our analysis are shown in Figure 7. The SPOD analysis of $S_{z}$ for V1 revealed the existence of two modes with a frequency of 0.15 mHz and one additional mode with a frequency of 0.24 mHz. In terms of their spatial structure, the first two modes correspond to the kink mode; however, they belong to the same eigenmode, just in different phase or polarisation (see the first two panels of the top row in Figure 7). Mode 6 has a morphology similar to an overtone sausage mode with a similar frequency to the fundamental sausage mode identified in IACD.

**Figure 8:**
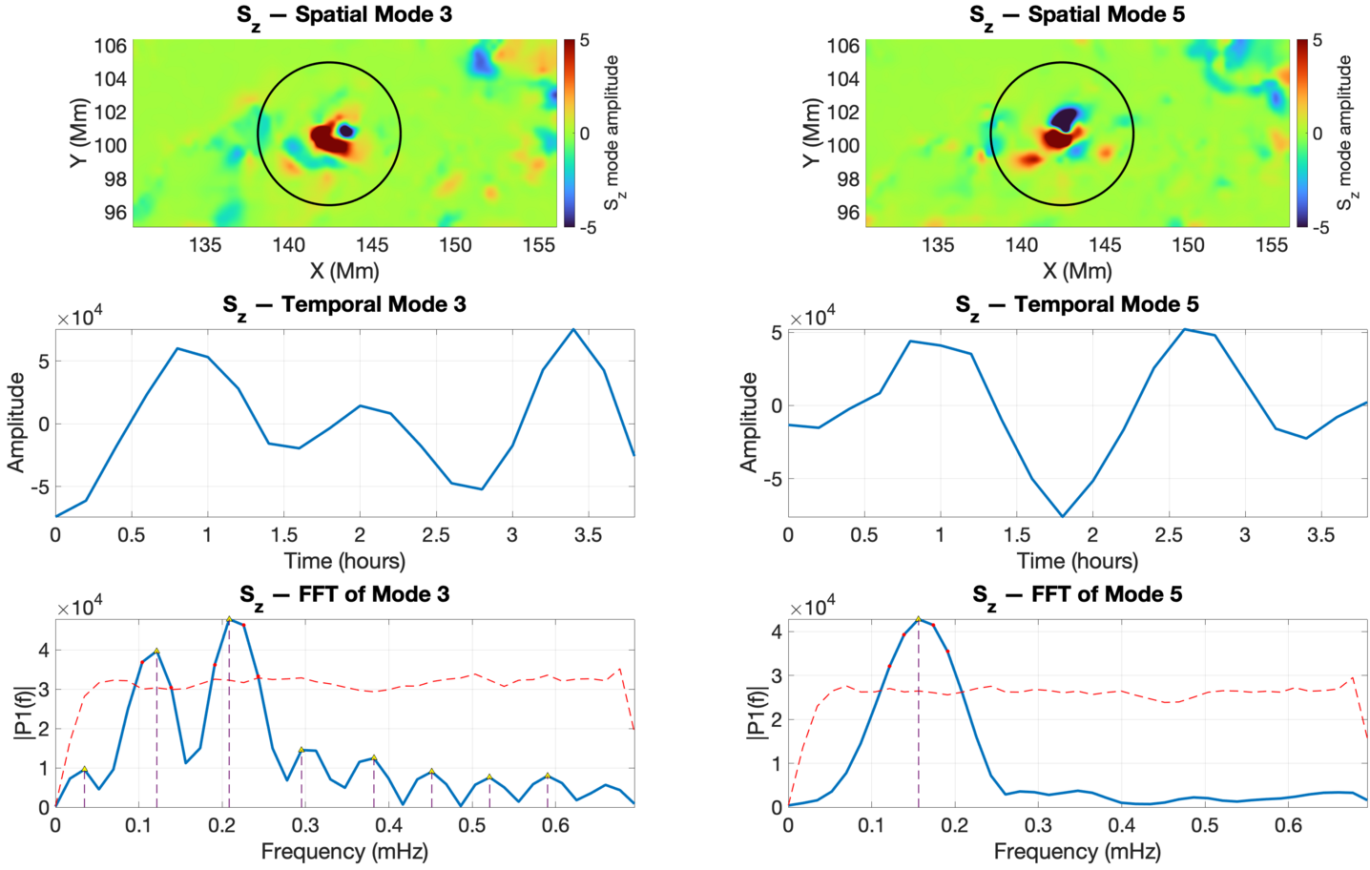
SPOD analysis of the vertical Poynting flux ($S_{z}$) in the region of the twisted magnetic flux-tube footpoint V2 during the interval 2011-02-13 19:00:00 UT – 22:48:00 UT. Top: spatial SPOD modes identified as wave modes; middle: corresponding temporal coefficients; bottom: FFT spectra of the temporal modes showing the dominant frequencies with 95% confidence thresholds.

In the case of the footpoint of the twisted magnetic flux tube V2, the SPOD analysis of the vertical Poynting flux is shown in Figure 8. In this case, the oscillatory behaviour is captured by spatial modes 3 and 5. Mode 3 exhibits peaks near 0.12 and 0.20 mHz, whereas mode 5 is dominated by a frequency around 0.16 mHz. The fact that we have two frequencies associated with mode 3 is likely due to other dynamics that were not properly filtered by SPOD. More precisely, the central peak in the temporal coefficient of mode 3 is smaller than the surrounding peaks, suggesting that it is unlikely to result from coherent wave activity. The associated high-frequency signal appears to be spurious, possibly arising from non-wave processes such as localised plasma motions or transient twisting of magnetic field lines. These effects can transiently enhance the Poynting flux, producing an apparent or “false” peak in the temporal coefficient that does not correspond to genuine MHD wave propagation. Moreover, the frequency implied by this central peak would be comparable to that of mode 5, which is inconsistent with the expected SPOD mode hierarchy, where lower modes typically represent lower-frequency, energetically dominant dynamics. Excluding this higher frequency for mode 3, it is more consistent with the expected ordering and therefore likely reflects the true physical oscillation.

### Analysis of the Vertical Component of the Magnetic Field

To assess the coupling between magnetic perturbations and vertical energy transport, we computed the SPOD of the vertical magnetic field component ($B_{z}$) time series for both ROIs of V1 and V2. This analysis provides direct information on the magnetic response of the twisted flux-tube system. When combined with the SPOD results from $S_{z}$ and current-based diagnostics (IACD), the $B_{z}$ analysis constrains the phase relation and relative energy content of the magnetic and kinetic components of the observed MHD waves.

**Figure 9:**
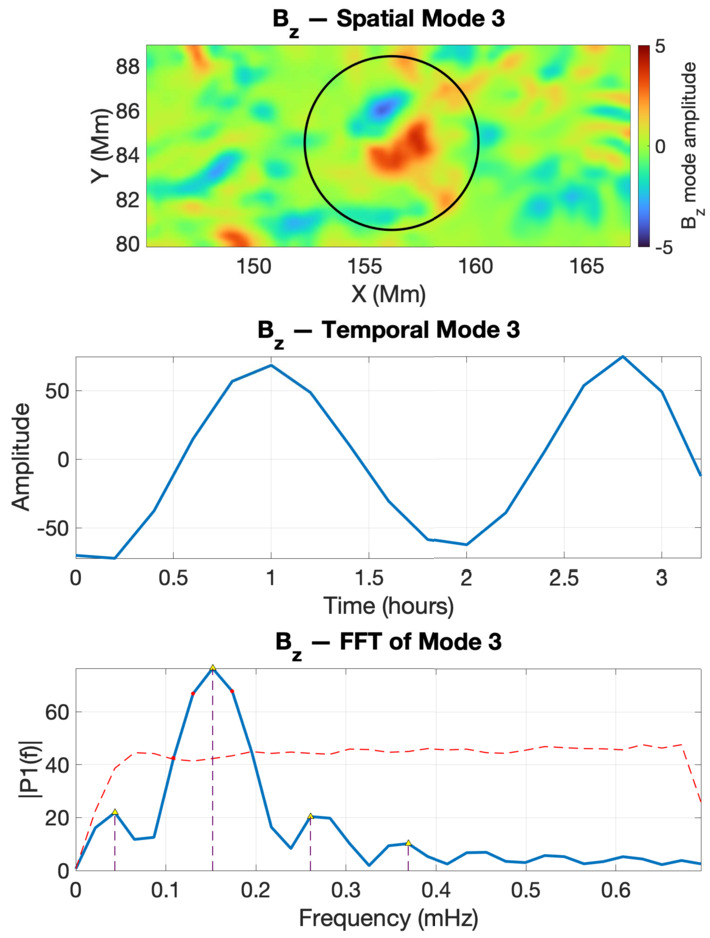
The same as in Figure 7, but here we show the SPOD analysis for the vertical magnetic field $B_{z}$ for the flux tube V1.

**Figure 10:**
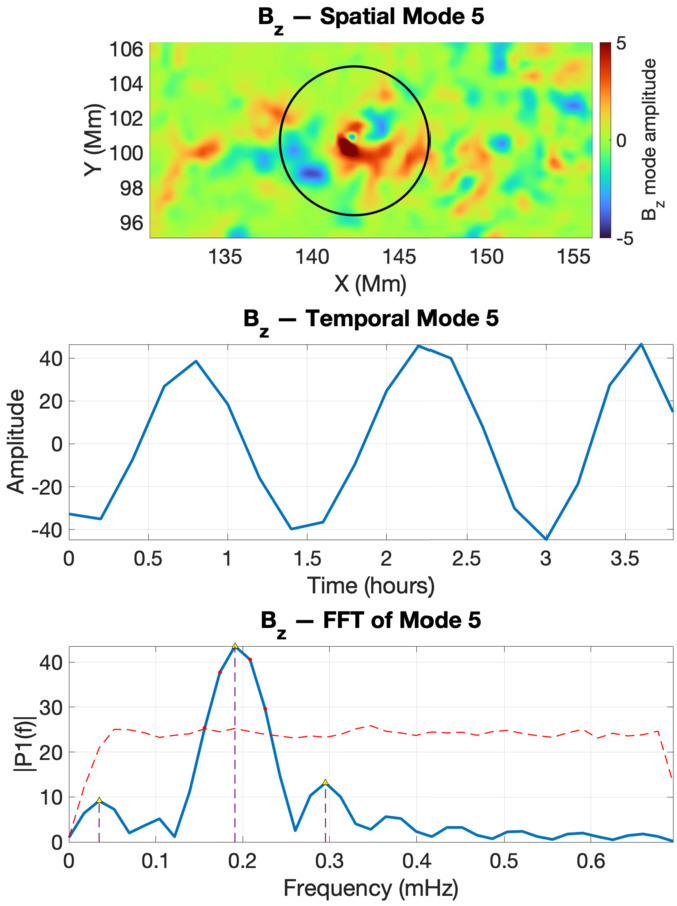
The same as in Figure 8, but here we show the SPOD analysis of the vertical magnetic field $B_{z}$ for the flux tube V2.

The SPOD analysis of $B_{z}$ for the twisted magnetic flux tubes V1 and V2 shows that the kink oscillation identified in other diagnostics is also present in the vertical magnetic field component (Figures 9 and 10). Interestingly, our analysis could not evidence the presence of the sausage mode. A plausible explanation is that the total magnetic flux through the tube remains approximately conserved, so the variations in $B_{z}$ tend to cancel out across the cross-section, resulting in a weaker net signal.

For V1, the measured frequency of the kink mode was 0.15 mHz and is identical to the values determined by the SPOD analysis in the IACD field and vertical Poynting flux. As for V2, the rotational kink was identified in IACD at a similar frequency, and it also appears in the $S_{z}$ modes as two orthogonal kink polarisations. The agreement between these diagnostics indicates that we are detecting a single kink oscillation that leaves consistent signatures in the magnetic field, current distribution, and vertical energy flux. The recurrence of the same frequency band in all three observables suggests that this kink mode is a stable and persistent feature of the flux tube dynamics, rather than being strongly influenced by local variations in stratification or geometric asymmetry.

**Figure 11:**
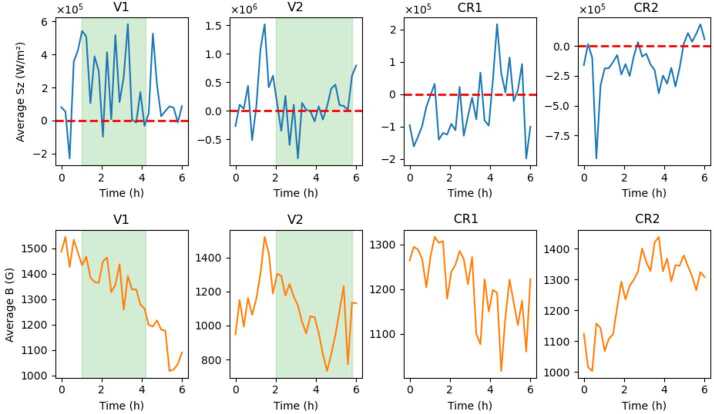
Time evolution of the spatially averaged vertical Poynting flux component $\langle S_{z} \rangle $ (top row) and the vertical magnetic field component $\langle B_{z} \rangle $ (bottom row) within four selected regions of interest (V1, V2, CR1 and CR2). The shaded green areas highlight the time intervals corresponding to the presence of a coherent twisted flux tube. The red dashed horizontal lines indicate $\langle S_{z} \rangle = 0$. The data are masked based on a threshold in the IACD to isolate significant regions: the twisted magnetic flux tube regions (V1 and V2) and the control regions exhibiting magnetic shear. Time is given in hours from the start of the observations.

Figure 11 shows the time evolution of the spatially averaged vertical Poynting flux component, $\langle S_{z} \rangle $ (top row), and the magnitude of the magnetic field, $\langle B \rangle $ (bottom row), in four selected regions of interest: V1, V2, and two control regions (Control Region 1 and 2). The time is given in hours from the start of the observations on 2011-02-13 17:00:00 UT. The control regions were selected based on the IACD field, specifically by choosing areas where the IACD exceeds 1, as such values indicate strong magnetic shear. In all ROIs, the data are masked using a threshold in the IACD field, restricting the analysis to regions with high magnetic twist or strong magnetic shear. The chosen threshold was IACD $\geq 33\%$ of the maximum IACD value in each region, providing similar-sized regions. The green shaded areas in Figure 11 indicate the time intervals where a coherent twisted flux tube is present, while the red dashed horizontal lines mark $\langle S_{z} \rangle = 0$. The flux tube regions (V1 and V2) show enhanced Poynting flux compared with the control regions, reflecting the energy transport associated with the twisted magnetic structures. The average of the Poynting flux for the temporal domain shown in Figure 11 shows that in the two flux tubes this value is $2.04\times 10^{5}$ Wm^−2^ and $1.75\times 10^{5}$ Wm^−2^, respectively, while the averages over the two control regions are $-4.68\times 10^{4}$ Wm^−2^ and $-1.28\times 10^{5}$ Wm^−2^. These values suggest that the twisted magnetic structures identified by our study (and structures that support waves) are the structures that allow the upward transport of energy contributing to the heating of the solar atmosphere. The mean values of $B_{z}$ suggest that the magnetic flux concentration in the analysed regions is similar, reinforcing that a twisted magnetic field is important for higher $S_{z}$.

The vertical Poynting flux associated with the twisted magnetic flux tube is more than sufficient to meet the local chromospheric heating requirement ($10^{5} - 10^{6}$ Wm^−2^). Even if only a few per cent of this flux is transmitted upward and dissipated, it could provide a significant contribution. However, these fluxes are estimated in the photosphere. Processes such as reflection, mode conversion, and dissipation will reduce the amount that actually reaches and heats the chromosphere. Assuming a typical flux tube diameter of 3 Mm, and based on the measured upward Poynting fluxes sustained by the footpoints of the twisted magnetic flux tubes, only a modest fractional coverage of about 0.4 – 4% of the photospheric surface by such structures would be sufficient to meet the average chromospheric heating requirement ($\sim 4\times 10^{3}~\mathrm{W\,m^{-2}}$). This corresponds to roughly $10^{3}$ – $10^{4}$ twisted magnetic flux tubes distributed over the solar surface.

## Conclusions

In this study, we investigated the oscillatory dynamics of the footpoints of twisted magnetic flux tubes in AR 11158. These twisted structures were identified using the IACD diagnostic applied to SHARP vector magnetograms, and their temporal behaviour was analysed with the SPOD method, which decomposes the complex dynamics of several key variables at the flux-tube footpoints: $B_{z}$, IACD, and $S_{z}$. By analysing these variables, we characterised the dominant MHD modes supported by the two most prominent twisted magnetic flux tubes within the analysed time interval.

Our results demonstrate that both structures exhibit an oscillatory behaviour consistent with kink and sausage MHD modes. Similar frequencies were obtained for kink modes recovered for all the analysed variables, confirming that these signatures correspond to genuine physical oscillations rather than artefacts of the decomposition. The close correspondence between the sausage-mode frequency in the IACD field (0.26 mHz) and the kink-like oscillation in the vertical Poynting flux (0.24 mHz) suggests that these oscillations are not independent but form part of a coupled kink–sausage mode system. In realistic, non-axisymmetric magnetic flux tubes, the presence of magnetic twist together with deviations from circular symmetry breaks the azimuthal mode degeneracy and promotes coupling between compressive ($m=0$) and transverse ($m=1$) perturbations, enabling energy exchange between sausage-like and kink-like motions. The Poynting flux, being sensitive to both velocity and magnetic perturbations, likely captures this coupling as a kink-like energy-flux pattern oscillating at the sausage frequency. The small frequency offset ($\approx 0.02$ mHz) reflects this weak mode splitting expected. The twisted magnetic flux tube V2 showed similar oscillatory behaviour at slightly lower frequencies (0.14 – 0.20 mHz), again displaying mixed-mode characteristics. The absence of a clear sausage-mode signature in $B_{z}$ does not imply that this mode is dynamically unimportant, but rather reflects the lower sensitivity of $B_{z}$ to axisymmetric compressive motions under our observing conditions. The sausage mode primarily modulates the cross-sectional area of the flux tube and the associated boundary currents, which leads to strong variations in magnetic gradients and current density and therefore produces a clear signal in IACD, as well as in the vertical Poynting flux $S_{z}$ through the coupling between velocity and magnetic perturbations. By contrast, the spatially averaged axial component $B_{z}$ is only weakly perturbed: for an approximately flux-conserving tube, radial expansion and contraction generate opposite-signed changes in $B_{z}$ across the cross-section that tend to cancel when integrated over the finite HMI resolution and along the line of sight. As a result, the sausage oscillation can remain clearly detectable in IACD and $S_{z}$, while its imprint in $B_{z}$ falls below the noise level.

The analysis of the vertical Poynting flux demonstrated that these twisted magnetic flux tubes are sites of highly variable, intermittent energy transport. Peak values of the upward-directed energy flux reach $10^{5}-10^{6}$ Wm^−2^, exceeding the average chromospheric heating requirement by one to two orders of magnitude. In terms of $B_{z}$, the mean values of this quantity suggest that the magnetic flux concentration in the analysed regions is similar, reinforcing that a twisted magnetic field is important for higher $S_{z}$.

Overall, our findings support the view that photospheric twisted magnetic flux tubes can guide, reflect, and couple multiple wave modes. These structures mediate localised energy transfer between magnetic and kinetic reservoirs and can channel energy upward into the higher layers of the solar atmosphere. The consistency of oscillatory frequencies across multiple observables (IACD, $B_{z}$, and $S_{z}$) provides robust observational evidence for MHD wave activity within these twisted magnetic flux concentrations. Future studies combining POD–based spectral methods with multi-height observations and advanced 3D numerical simulations will be essential for tracing how twisted magnetic flux tubes guide MHD waves and channel energy dissipation through the chromosphere and into the corona.

## Data Availability

No datasets were generated during the current study.

## Footnotes

1 https://github.com/DavidMacT/ARTop.
